# Expression of nicotinamide N-methyltransferase in hepatocellular carcinoma is associated with poor prognosis

**DOI:** 10.1186/1756-9966-28-20

**Published:** 2009-02-16

**Authors:** Jongmin Kim, Seok Joo Hong, Eun Kyung Lim, Yun-Suk Yu, Seung Whan Kim, Ji Hyeon Roh, In-Gu Do, Jae-Won Joh, Dae Shick Kim

**Affiliations:** 1 Cbs Bioscience Inc., 59-5 Jang-Dong, Yuseong-gu, Daejeon, 305-343, Korea; 2 Department of Emergency Medicine, Chungnam National University Hospital, Daejeon, 301-721, Korea; 3 Department of Pathology, Samsung Medical Center, Sungkyunkwan University School of Medicine, Seoul, 135-710, Korea; 4 Department of Surgery, Samsung Medical Center, Sungkyunkwan University School of Medicine, Seoul, 135-710, Korea

## Abstract

**Background:**

Hepatocellular carcinoma (HCC) is the most common tumor in the adult liver, with high relapse and mortality rates despite diverse treatment modalities. In this study, nicotinamide N-methyltransferase (NNMT), a key enzyme in drug metabolism, was investigated as a potential prognostic factor.

**Methods:**

Frozen tumors and non-cancerous surrounding tissues from 120 patients with primary HCC were studied. Expressions of NNMT and internal control genes were measured by real-time reverse-transcription PCR (RT-PCR). The relationship of NNMT mRNA level with clinicopathologic parameters and clinical outcome was evaluated.

**Results:**

NNMT mRNA level is markedly reduced in HCCs compared to non-cancerous surrounding tissues (P < 0.0001), and NNMT expression in tumors was significantly correlated with tumor stage (P = 0.010). Moreover, stratification of patients based on tumor NNMT mRNA levels revealed that the patients who expressed higher NNMT mRNA levels tended to have a shorter overall survival (OS) time (P = 0.053) and a significantly shorter disease-free survival (DFS) time (P = 0.016). Both NNMT expression (P = 0.0096) and tumor stage (P = 0.0017) were found to be significant prognostic factors for DFS in a multivariate analysis.

**Conclusion:**

The results of this study indicated that NNMT gene expression is associated with tumor stage and DFS time in HCC cases. Because of the broad substrate specificity of NNMT, which could alter the efficacy and adverse effects of chemotherapy, NNMT merits further investigation regarding its role as a prognostic factor with a larger cohort of HCC patients.

## Background

Hepatocellular carcinoma (HCC) is the fifth most common cancer worldwide and the most common form of liver cancer, being responsible for 80% of primary malignant tumors in adults. HCC causes more than 600,000 deaths annually worldwide [1] and its endemic prevalence in Asia, including South Korea, makes HCC one of the top causes of death in this region. HCC is a type of tumor that is highly resistant to available chemotherapeutic agents, administered either alone or in combination [2]. Thus, in many cases, no effective therapy can be offered to patients with HCC. Therefore, it is of vital importance to identify important prognostic factors and novel molecular targets of HCC to develop targeted therapies, ultimately advancing therapeutic strategies of HCC in general.

Current evidence indicates that the precancerous liver and the early stages in HCC development are characterized by certain common traits governed by both genetic and epigenetic mechanisms [3,4]. These include the alteration of numerous signaling pathways leading to autonomous and deregulated cell proliferation and resistance to cell death [4-7]. Therefore, it is important to better understand the roles of deregulated genes in hepatocellular carcinogenesis. Derangements in various methylation processes in liver diseases have been identified [8,9], including increased nicotinamide methylation in cirrhotic patients [10]. Nicotinamide N-methyltransferase (NNMT) catalyzes the N-methylation of nicotinamide, pyridines, and other structural analogues [11]. It is involved in the biotransformation of many drugs and xenobiotic compounds. Although several studies indicated differential expression of NNMT in HCC specimens [12-15], the clincopathologic relevance of NNMT expression has not been fully investigated.

The aim of the present investigation was to examine whether NNMT expression could be used to predict the clinical course of HCC. Using a real-time RT-PCR analysis of NNMT gene expression, we found significant correlation between NNMT mRNA levels and poor prognosis of HCC. Thus, potential biological changes related to NNMT gene expression require further study, as they may have implications in predicting clinical outcome and choosing treatment modalities, due to the central role of NNMT in biotransformation and detoxification.

## Methods

### Patients and tissue samples

HCC (T) and corresponding non-cancerous hepatic tissues (NT) were obtained with informed consent from 120 patients who underwent curative hepatectomy for primary HCC between 2001 and 2006 in the Department of Surgery, Samsung Medical Center, Korea. The study protocol was approved by the Institutional Review Board of Samsung Medical Center. Complete clinical data were available in all 120 cases (median follow-up, 50 months; range, 3 – 92 months). The patients, ranging in age from 21 to 78 years (mean, 51.3 years) and having adequate liver function reserve, had survived for at least 2 months after hepatectomy, and none received treatment prior to surgery such as transarterial chemoembolization or radiofrequency ablation. Clinicopathologic features of the 120 HCCs in this study are described in Table 1. Surgically resected specimens were partly embedded in paraffin after fixation in 10% formalin for histological processing and partly immediately frozen in liquid nitrogen and stored at -80°C. All available hematoxylin and eosin stained slides were reviewed. The tumor grading was based on the criteria proposed by Edmondson and Steiner (I, well differentiated; II, moderately differentiated; III, poorly differentiated; IV, undifferentiated) [16]. The conventional TNM system outlined in the cancer staging manual (6th ed.) by the American Joint Committee on Cancer (AJCC) was used in tumor staging.

**Table 1:** Relations between NNMT mRNA levels and clinicopathologic features in HCC

	All patients (n = 120)		
		
Clinicopathologic parameters	High NNMT (n = 48) Copy number ratio ≥ 4.40	Low NNMT (n = 72) Copy number ratio < 4.40	P value
Age			0.730

< 55 years	31	43	

≥ 55 years	17	29	

Gender			0.758

Male	38	54	

Female	10	18	

HbsAg			0.885

Absent	8	14	

Present	40	58	

HCV			0.823

Absent	45	67	

Present	3	5	

Liver cirrhosis			0.852

Absent	25	40	

Present	23	32	

Tumor stage			0.010

I	23	23	

II	9	33	

III & IV	16	16	

AFP level			0.314

< 100 ng/ml	28	34	

≥ 100 ng/ml	20	38	

Tumor size			0.733

< 5 cm	27	44	

≥ 5 cm	21	28	

Edmondson grade			0.368

I	13	15	

II	30	43	

III & IV	5	14	

### RNA extraction and cDNA synthesis

Total RNA was extracted from cancerous and surrounding non-cancerous frozen tissues using an RNeasy minikit (Qiagen, Germany) according to the manufacturer's instructions. The integrity of all tested total RNA samples was verified using a Bioanalyzer 2100 (Agilent Technologies, United States). DNase I treatment was routinely included in the extraction step. Residual genomic DNA contamination was assayed by a quantitative real-time PCR assay for GAPDH DNA and samples with contaminating DNA were re-subjected to DNase I treatment and assayed again. Samples containing 4 μg of total RNA were incubated with 2 μl of 1 μM oligo d(T)_18 _primer (Genotech, Korea) at 70°C for 7 min and cooled on ice for 5 min. The enzyme mix was separately prepared in a total volume of 11 μl by adding 2 μl of 0.1 M DTT (Duchefa, Netherlands), 2 μl of 10× reverse-transcription buffer, 5 μl of 2 mM dNTP, 1 μl of 200 U/μl MMLV reverse-transcriptase, and 1 μl of 40 U/μl RNase inhibitor (Enzynomics, Korea). After adding the enzyme mix to the annealed total RNA sample, the reaction was incubated for 90 min at 42°C prior to heat inactivation of reverse-transcriptase at 80°C for 10 min. The cDNA samples were brought up to a final volume of 400 μl by the addition of diethylpyrocarbonate (DEPC)-treated water.

### Quantitative real-time PCR

Real-time PCR amplifications were carried out in 384 well plates according to the instructions of the manufacturer, using Applied Biosystems PRISM 7900HT instruments. The real-time PCR analysis was performed in a total volume of 10 μl with 5 μl of 2× Taqman gene expression master mix (Applied Biosystems, United States), 1 μl each of 5 μM forward and reverse primers and 1 μM probe (Genotech), and 2 μl of cDNA (or water as a control, which was always included). The amplification steps were as follows: an initial denaturation step, 95°C for 10 min, followed by 40 cycles of denaturation at 95°C for 15 sec; elongation at 60°C for 1 min. The primer and probe sequences were designed using Primer Express 3.0 software (Applied Biosystems) and all probe sequences were labeled with FAM at the 5' end and with TAMRA at the 3' end. The following primer and probe sequences were used: B2M forward (5'-CAT TCG GGC CGA GAT GTC T-3'), reverse (5'-CTC CAG GCC AGA AAG AGA GAG TAG-3') and probe (5'-CCG TGG CCT TAG CTG TGC TCG C-3'); GAPDH forward (5'-CAC ATG GCC TCC AAG GAG TAA-3'), reverse (5'-TGA GGG TCT CTC TCT TCC TCT TGT-3') and probe (5'-CTG GAC CAC CAG CCC CAG CAA G-3'); HMBS forward (5'-CCA GGG ATT TGC CTC ACC TT-3'), reverse (5'-AAA GAG ATG AAG CCC CCA CAT-3') and probe (5'-CCT TGA TGA CTG CCT TGC CTC CTC AG-3'); HPRT1 forward (5'-GCT CGA GAT GTG ATG AAG GAG AT-3'), reverse (5'-CCA GCA GGT CAG CAA AGA ATT-3') and probe (5'-CCA TCA CAT TGT AGC CCT CTG TGT GCT C-3'); SDHA forward (5'-CAC CTA GTG GCT GGG AGC TT-3'), reverse (5'-GCC CAG TTT TAT CAT CTC ACA AGA-3') and probe (5'-TGG CAC TTA CCT TTG TCC CTT GCT TCA-3'); NNMT forward (5'-TTG AGG TGA TCT CGC AAA GTT ATT-3'), reverse (5'-CTC GCC ACC AGG GAG AAA-3') and probe (5'-CCA CCA TGG CCA ACA ACG AAG GAC-3'). Expression of NNMT mRNA was measured (the number of cycles required to achieve a threshold, or C_T_) in triplicate, and then normalized relative to a set of reference genes (B2M, GAPDH, HMBS, HPRT1, and SDHA) by subtracting the average of the expression of the 5 reference genes [17]. Using the ΔC_T _value (NNMT C_T _- average C_T _of reference genes), the mRNA copy number ratio was calculated as 2^-ΔCt^. Standard curves were constructed from the results of simultaneous amplifications of serial dilutions of the cDNA samples.

### Statistical analysis

All statistical analyses were done with the open source statistical programming environment R . Significant differences between gene expression levels were evaluated by a Student's t test. Correlation between gene expression and clinicopathologic variables was evaluated using a χ^2 ^test. Categorical clinicopathologic variables were classified as in another study on HCC prognosis [18], and continuous clinicopathologic variables were classified by cutoff values close to their medians as in other studies [19,20]. For instance, the cutoff value of 100 ng/ml AFP level has been used in another study [19], and the cutoff values of 52 and 56 years have been used in other recent studies [18,20]. Kaplan-Meier survival curves were calculated using tumor recurrence (defined as the first appearance of a tumor at any site following definitive treatment) or death as the end points. The difference of overall survival curve or disease-free survival curve was examined by log-rank test. In addition, the Cox proportional hazard regression model was used to identify independent prognostic factors for overall survival and disease-free survival. A two-tailed P value test was used and a P value of < 0.05 was considered statistically significant.

## Results

### Expression of NNMT gene in hepatocellular carcinoma

**Figure 1:**
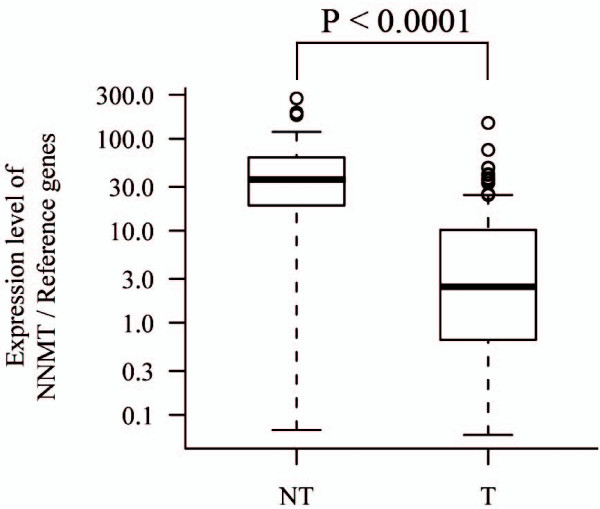
**Box and whiskers plot for NNMT mRNA levels in non-cancerous liver (NT) and HCC (T) determined by real-time RT-PCR**. The box is marked by the first and third quartile with the median marked by a thick line. The whiskers extend to the most extreme data point which is no more than 1.5 times the interquartile range from the box.

We performed real-time RT-PCR for NNMT mRNA from frozen paired samples derived from 120 patients with HCC. A total of 120 HCCs (T) and 40 non-cancerous hepatic samples (NT) were assessed by real-time RT-PCR. Expression of NNMT mRNA was measured in triplicate, and then normalized relative to a set of reference genes (B2M, GAPDH, HMBS, HPRT1, SDHA) by subtracting the average of the expression of the 5 reference genes [17]. NNMT mRNA was significantly lower in T than in NT tissues (2.47 vs 35.75; median copy number ratio, P < 0.0001) (Figure 1). The reduced expression of NNMT mRNA in HCC is consistent with findings of other studies including research employing microarray measurements [12-15]. In addition, NNMT mRNA was higher in recurrent tumors than in non-recurrent tumors (3.93 vs 1.56; median copy number ratio, P = 0.21), especially in stage III & IV tumors (7.26 vs 0.95; median copy number ratio, P = 0.056), although the differences were not statistically significant (data not shown).

### Relationship between tumor NNMT mRNA level and clinicopathologic features

To better understand the significance of NNMT expression in HCC, we correlated the mRNA expression level with the major clinicopathologic features. The statistically most significant cutoff value of NNMT mRNA level discriminating between patients with a good prognosis and patients with a poor prognosis was used. As shown in Table 1, NNMT expression was significantly associated with tumor stage (P = 0.010) in 120 HCCs. However, no correlation was observed between NNMT mRNA level and other clinicopathologic parameters (age, gender, virus, liver cirrhosis, tumor size, Edmondson grade, and AFP level) (P > 0.05).

### Impact of tumor NNMT mRNA levels on OS and DFS

**Figure 2:**
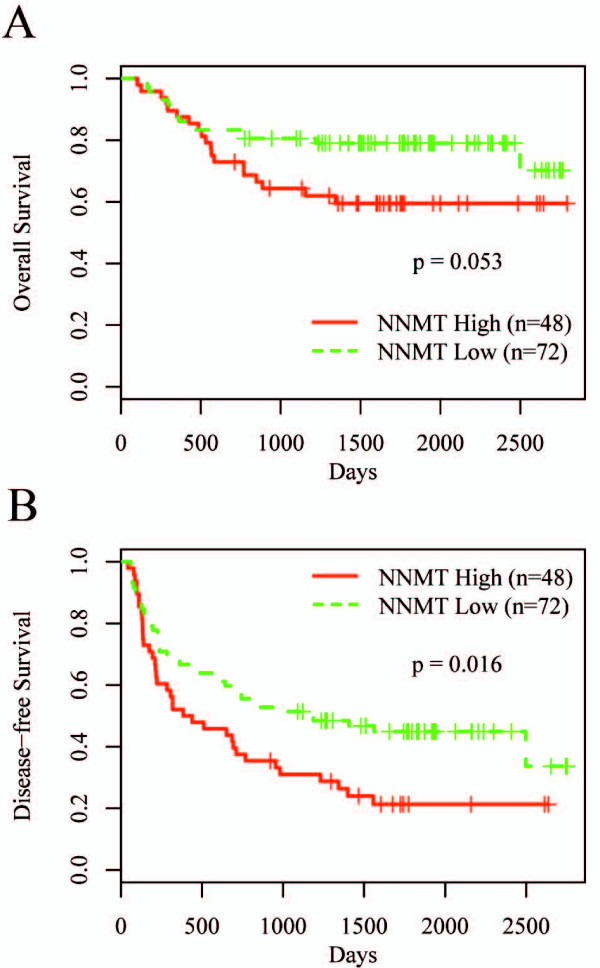
**Kaplan-Meier curves for OS and DFS of patients with high and low NNMT mRNA levels after surgery**. A, patients with high NNMT mRNA levels (≥ 4.40; copy number ratio) tended to have a shorter OS time (P = 0.053). Broken lines, patients with low NNMT mRNA levels (n = 72); thin lines, patients with high NNMT mRNA levels (n = 48). B, patients with high NNMT mRNA levels had a significantly shorter DFS time (P = 0.016). Broken lines, patients with low NNMT mRNA levels (n = 72); thin lines, patients with high NNMT mRNA levels (n = 48).

During the follow-up observation period of up to 92 months, locoregional recurrence or distant metastases occurred in 72 patients (60%) and death was confirmed in 35 patients (29%). To assess the prognostic significance of NNMT expression, we analyzed overall survival (OS) and disease-free survival (DFS) rates using the Kaplan-Meier method. At the 5-year follow-up, approximately 79% of the patients with low NNMT expression (< 4.40; copy number ratio) survived, whereas 60% of the patients with high NNMT expression (≥ 4.40; copy number ratio) survived (Figure 2A). Similarly, at the 5-year follow-up, approximately 45% of the patients with low NNMT expression were disease-free, whereas 22% with high NNMT expression were disease-free (Figure 2B). The log-rank test showed that patients who expressed higher NNMT mRNA levels tended to have a shorter OS time (P = 0.053) and a significantly shorter DFS time (P = 0.016). A univariate Cox regression analysis was used to identify important prognostic factors of OS and DFS. High Edmondson grade (grade I vs II, P = 0.020; grade I vs III-IV, P = 0.019), high AFP level (P = 0.0070), large tumor size (P = 0.00012), and high tumor stage (stage I vs II, P = 0.0068; stage I vs III-IV, P = 2.2 × 10^-5^) were identified as important risk factors for OS (Table 2), whereas high NNMT mRNA level (P = 0.018) and high tumor stage (stage I vs III-IV, P = 0.0049) were identified as important risk factors for DFS (Table 3). In a multivariate Cox analysis, both NNMT expression (P = 0.0096) and tumor stage III & IV (P = 0.0017) were found to be significant prognostic factors for DFS (Table 4).

**Table 2:** Univariate Cox regression analysis for overall survival

Variable	Hazard Ratio	95% Confidence Interval		P value
			
		Lower limit	Upper limit	
Age (< 55 years vs ≥ 55 years)	0.76	0.38	1.53	0.45

Gender (male vs female)	1.00	0.46	2.21	1.00

Edmondson grade (I vs II)	5.51	1.31	23.2	0.02

Edmondson grade (I vs III – IV)	6.53	1.36	31.4	0.019

HbsAg (absent vs present)	1.49	0.58	3.83	0.41

HCV (absent vs present)	2.06	0.73	5.87	0.17

AFP level (< 100 ng/ml vs ≥ 100 ng/ml)	2.67	1.31	5.46	0.0070

Liver cirrhosis (absent vs present)	1.50	0.77	2.93	0.23

Tumor size (< 5 cm vs ≥ 5 cm)	4.07	1.99	8.31	0.00012

Tumor stage (I vs II)	7.81	1.76	34.6	0.0068

Tumor stage (I vs III – IV)	23.5	5.48	100.9	2.2 × 10^-5^

NNMT (low vs high)	1.91	0.98	3.71	0.057

**Table 3:** Univariate Cox regression analysis for disease-free survival

Variable	Hazard Ratio	95% Confidence Interval		P value
			
		Lower limit	Upper limit	
Age (< 55 years vs ≥ 55 years)	0.80	0.50	1.27	0.34

Gender (male vs female)	1.02	0.60	1.73	0.95

Edmondson grade (I vs II)	1.25	0.72	2.17	0.43

Edmondson grade (I vs III – IV)	1.08	0.50	2.30	0.85

HbsAg (absent vs present)	1.02	0.58	1.80	0.94

HCV (absent vs present)	2.11	0.97	4.60	0.061

AFP level (< 100 ng/ml vs ≥ 100 ng/ml)	1.19	0.76	1.86	0.45

Liver cirrhosis (absent vs present)	1.14	0.73	1.78	0.57

Tumor size (< 5 cm vs ≥ 5 cm)	1.30	0.82	2.04	0.26

Tumor stage (I vs II)	1.10	0.64	1.89	0.72

Tumor stage (I vs III – IV)	2.22	1.27	3.87	0.0049

NNMT (low vs high)	1.72	1.10	2.70	0.018

**Table 4:** Multivariate Cox regression analysis for disease-free survival

Variable	Hazard Ratio	95% Confidence Interval		P value
			
		Lower limit	Upper limit	
NNMT (low vs high)	1.89	1.17	3.07	0.0096

Tumor stage (I vs II)	1.42	0.80	2.54	0.23

Tumor stage (I vs III – IV)	2.47	1.40	4.33	0.0017

## Discussion

The metabolism of drugs, toxic chemicals, and hormones is important in the fields of pharmacology and endocrinology given its implication in many pathophysiological processes, such as cancer and resistance to chemotherapy [21]. One of the key enzymes involved in biotransformation and drug metabolism is NNMT, which catalyzes the N-methylation of nicotinamide, pyrimidines, and other structural analogues [22,23]. NNMT is predominantly expressed in the liver, where its activity varies with a bimodal frequency distribution, thus raising the possibility that a genetic polymorphism might play a role in regulating the enzyme activity [23]. Lower expression is observed in other organs such as the kidney, lungs, placenta, heart, and brain. Although several studies indicated differential expression of NNMT in HCC [12-15], the role of NNMT in the molecular pathogenesis of HCC has yet to be elucidated.

This study focused on NNMT as a potential molecular marker responsible for determining clinicopathologic features and the prognosis of HCC. Utilizing a large number of HCC specimens, the quantitative real-time PCR assay showed that the expression of NNMT is markedly reduced in HCCs compared to non-cancerous surrounding tissues, consistent with other studies [12-15]. Stratification of HCC specimens based on NNMT gene expression levels showed that NNMT expression was significantly correlated with tumor stage (P = 0.010). More importantly, the log-rank test showed that patients who expressed higher NNMT mRNA levels tended to have a shorter OS time (P = 0.053) and a significantly shorter DFS time (P = 0.016). Both NNMT expression (P = 0.0096) and high tumor stage (P = 0.0017) were found to be significant prognostic factors for DFS in a multivariate analysis. It is not clear why NNMT expression level was a significant prognostic factor for DFS but not for OS. We believe that the limited follow-up time was not the main cause of lack of correlation between NNMT and OS because the events (death or relapse) were rare after the median follow-up time of 50 months in our cohort. Our analysis of NNMT expression in correlation with the clinicopathologic features and prognosis of HCC yielded the novel finding that NNMT mRNA levels could be used as a prognostic factor for DFS.

The mechanism for reduced expression of NNMT and its relation to HCC progression is not clear. Several metallothionein genes involved in detoxification and drug metabolism are downregulated in HCC especially in tumors with high Edmonson grades, reflecting de-differentiation of cancer cells [12]. Thus, it is possible that the liver specific function of NNMT is lost during the progression of HCC. On the other hand, a recent in vitro study found that NNMT was necessary for cancer cell migration in bladder cancer cell lines [24], pointing to a possible involvement in tumor invasion. In 120 HCCs observed in this study, NNMT mRNA was higher in recurrent tumors than in non-recurrent tumors especially in stage III & IV tumors, although the differences were not statistically significant. Thus, there's a possibility that increased NNMT expression is related to cell mobility and tumor invasiveness in high stage HCC. Interestingly, the NNMT expression level was decreased in stage II tumors compared to stage I tumors, while stage III & IV tumors showed a similar NNMT level as stage I tumors. This could be due to tumor de-differentiation preceding tumor invasion. However, we cannot rule out other regulatory mechanisms independent of tumor de-differentiation and invasion.

In tumors, abnormal expression of NNMT has been reported in glioblastoma [25], stomach cancer [26,27], papillary thyroid cancer [28,29], colon cancer [30], and renal carcinoma [31,32]. NNMT was identified as a novel serum marker for human colorectal cancers although this protein is not thought to be secreted [30]. Interestingly, the upregulation of NNMT was found to be inversely correlated with tumor size in renal clear cell carcinoma, suggesting that the enzyme may be significant in an initial phase of malignant conversion [32]. Increased expression of NNMT in non-tumor cells was reported in a few situations: the cerebellum of patients with Parkinson's disease [33,34], human hepatoma cells (Huh7) with expression of the hepatitis C core protein [35], and the liver of mice transplanted with tumors [36,37]. In these situations, the mechanism for deregulated NNMT expression remains unclear.

Recently, NNMT promoter was cloned and studied in papillary thyroid cancer cell lines, where it was shown to be activated by hepatocyte nuclear factor-1β [29]. Subsequently, it was found that the NNMT promoter region also contains the consensus sequences for signal transducers and activators of transcription (STAT) binding elements and nuclear factor-interleukin (IL) 6 binding elements [38]. Accordingly, hepatoma cell line (Hep-G2), which expressed low levels of NNMT, increased NNMT expression several fold upon stimulation by IL-6. The stimulation by IL-6 was largely abolished with the expression of dominant-negative STAT3 [38]. Activation of STAT3 alone caused a four-fold higher induction of NNMT promoter activity in the transformed Hep-G2 cells. Thus, NNMT expression could be regulated by IL-6 and STAT3 in a subclass of HCC. The expression of NNMT analyzed in relation to the expression of related regulatory molecules could improve the predictive power on HCC prognosis.

To our knowledge, this is the first report of NNMT as a prognostic factor of DFS in HCC. The findings herein indicate that NNMT is an attractive target for therapeutic regulation because it is involved in drug metabolism and could alter the efficacy of standard chemotherapeutic drugs. Additional research in larger populations of HCC patients may ultimately determine the ability of NNMT in accurate diagnosis and sub-classification of HCC.

## Conclusion

We found that NNMT was associated with the tumor stage and that higher NNMT mRNA levels in HCC was significantly associated with shorter DFS time. It is very important to develop new target molecules and to establish novel chemotherapy strategies in malignancies such as HCC, which shows frequent relapse and high mortality despite various treatment modalities. The broad substrate specificity of NNMT suggests that it could alter the efficacy and/or adverse effect of standard doses of chemotherapeutic drugs. Therefore, NNMT merits further study for its role as a prognostic factor of OS and DFS with a larger cohort of HCC patients. Moreover, NNMT itself could be a target for chemotherapeutic agents. Establishing the molecular interactions of NNMT with diverse molecular pathogenic factors in HCC will enable new studies and development of effective therapeutic regimens.

## Abbreviations

HCC: hepatocellular carcinoma; NNMT: nicotinamide N-methyltransferase; RT-PCR: reverse-transcription PCR; OS: overall survival; DFS: disease-free survival; T: hepatocellular carcinoma tissue samples; NT: non-cancerous hepatic tissue samples; STAT: signal transducers and activators of transcription; IL: interleukin.

## Competing interests

The authors declare that they have no competing interests.

## Authors' contributions

JK analyzed the RT-PCR data and wrote the manuscript. SH and SK helped write the paper. EL and YY carried out the RT-PCR experiment. JR and ID collected the samples and patients' clinical data. JJ analyzed patients' clinical data and helped write the final version. DK conceived of the study and wrote the manuscript. All authors read and approved the final manuscript.
